# An unusual new species of paguroid (Crustacea, Anomura, Paguridae) from deep waters of the Gulf of Mexico

**DOI:** 10.3897/zookeys.449.8541

**Published:** 2014-10-22

**Authors:** Rafael Lemaitre, Ana Rosa Vázquez-Bader, Adolfo Gracia

**Affiliations:** 1 Department of Invertebrate Zoology, National Museum of Natural History, Smithsonian Institution, 4210 Silver Hill Road, Suitland, MD 20746, USA; 2 Laboratorio de Ecología Pesquera de Crustáceos, Instituto de Ciencias del Mar y Limnología, UNAM, Av. Universidad # 3000, Universidad Nacional Autónoma de México, CU, Distrito Federal, 04510, México

**Keywords:** *Tomopaguropsis*, new species, Paguridae, hermit crab, deep water, Gulf of Mexico

## Abstract

A new hermit crab species of the family Paguridae, *Tomopaguropsis ahkinpechensis* **sp. n.**, is described from deep waters (780–827 m) of the Gulf of Mexico. This is the second species of *Tomopaguropsis* known from the western Atlantic, and the fifth worldwide. The new species is morphologically most similar to a species from Indonesia, *Tomopaguropsis crinita* McLaughlin, 1997, the two having ocular peduncles that diminish in width distally, reduced corneas, dense cheliped setation, and males lacking paired pleopods 1. The calcified figs on the branchiostegite and anterodorsally on the posterior carapace, and the calcified first pleonal somite that is not fused to the last thoracic somite, are unusual paguroid characters. A discussion of the affinities and characters that define this new species is included, along with a key to all five species of *Tomopaguropsis*.

## Introduction

Few deep ocean basins have been sampled or studied more intensely than the Gulf of Mexico. From the late 1800s to the 1940s, only a handful of stations had been sampled in the Gulf, primarily during cruises of the US Coast and Geodetic Survey’s steamer *Blake*, US Fish Commission Steamer *Albatross*, HP Bingham’s yacht *Pawnee I*, and Woods Hole Oceanographic Institution’s R/V *Atlantis* (Bayer 1969, Pequegnat and Chace 1970). Since the 1950s, however, the Gulf has been crisscrossed by numerous exploratory or oceanographic expeditions, particularly on board the R/V *Oregon*, R/V *Oregon* II, M/V *Silver Bay*, M/V *Combat*, R/V *Pelican*, R/V *Pillsbury*, R/V *Alaminos*, and B/O *Justo Sierra* (Bullis and Thompson 1965, Pequegnat and Chace 1970, Vázquez-Bader and Gracia 2013, Vázquez-Bader et al. 2014), which altogether have accumulated in museums and research institutions a vast collection of invertebrates discussed in numerous taxonomic reports (see summary in Felder and Camp 2009). Yet, new species are still being discovered in deep waters of the Gulf, as documented in this paper with the description of a new hermit crab of the family Paguridae.

During the 2011 and 2013 cruises of the B/O *Justo Sierra* of the Universidad Nacional Autónoma de México (UNAM), as part of studies of the deep water (300–1200 m) benthic communities and fishery resources from the Mexican coast, two male specimens of an unusual species of hermit crab were collected in deep water near the Campeche Bank. A study of these relatively large specimens (about 65 mm in stretched body length) showed they represent an undescribed species assignable to *Tomopaguropsis* Alcock, 1905, a genus previously known to be represented in the western Atlantic by a single species only, *Tomopaguropsis problematica* (A. Milne-Edwards & Bouvier, 1893). McLaughlin (1997) discussed and expanded *Tomopaguropsis* with the description of two new species from Indonesian waters, *Tomopaguropsis crinita* McLaughlin, 1997, and *Tomopaguropsis miyakei* McLaughlin, 1997, and selected *Tomopaguropsis lanata* Alcock, 1905, from the Indian Ocean, as the type species of the genus, although McLaughlin (1997: 546, 552; 2003: 128) consistently misspelled the type species name as “*Tomopaguropsis lantana*”. In addition to their deep water habitat, usually ranging from continental shelf to upper slope depths (about 100 to 750 m), the previously known species of this genus are characterized primarily by having 13 pairs of quadriserial gills; crista dentata with accessory tooth; chelipeds subequal (right slightly more robust); fourth pereopod with propodal rasp consisting of several rows of corneous scales, dactyl with or without preungual process; and males and females with unpaired left pleopods 2–5, males often but not always, with paired small pleopods 1 modified as gonopods. The new species of *Tomopaguropsis* is fully described, and its affinities with other congeners and some unusual characters of phylogenetic significance, are discussed.

## Materials and methods

The holotype of the new species described herein is deposited in the collections of the Laboratorio de Ecología Pesquera de Crustáceos, Instituto de Ciencias del Mar y Limnología, UNAM (EPC), and a paratype in the National Museum of Natural History, Smithsonian Institution, Washington DC (USNM). The specimens were collected during deep-water cruises of the B/O *Justo Sierra*, using a commercial shrimp trawl with an 18 m opening, equipped with a net having 4.5 cm mesh size along the body, and 1.5 cm mesh size on the cod end. Each trawl lasted for 30 min at a ship speed of 2.5 to 3.0 knots. Samples were sorted and preserved in 70% ethanol and transported to the Laboratorio de Ecología Pesquera de Crustáceos of Instituto de Ciencias del Mar y Limnología, UNAM, for study.

General terminology follows that used by McLaughlin and Lemaitre (2001), with specialized terminology for the lines and calcified portions of branchiostegite and posterior carapace after Tudge et al. (2012). Pereopods and pleopods are indicated with a number, except for chelipeds which correspond to pereopods 1. Measurements indicated for the specimens are of shield length, measured in millimeters (mm) from the midpoint of rostral lobe to midpoint of posterior margin of shield. Abbreviations: B/O, buque oceanográfico; COBERPES, Comunidades Bentónicas y Recursos Pesqueros Potenciales del Mar Profundo del Golfo de México; R/V, research vessel; sta, station.

## Taxonomy

### Family Paguridae Latreille, 1802

#### Tomopaguropsis ahkinpechensis sp. n.

Taxon classificationAnimaliaDecapodaPaguridae

http://zoobank.org/411C7597-63C1-4085-9748-FEC4CDFEFFBC

Figs 1 , 2 , 3 , 4

##### Type material.

Holotype: COBERPES 3, sta α10, 19°40.066N, 92°45.490W, 780 m, 19 November 2011: 1 male 8.9 mm (EPC 201310). Paratype: COBERPES 5, sta A11, 19°32.108N, 93°08.373W, 827 m, 24 May 2013: 1 male 8.1 mm (USNM 12376795).

##### Description.

Thirteen pairs of quadriserial gills. Arthrobranchs on third maxilliped and somite X (thoracomere 4, or cheliped) well developed. Pleurobranchs well developed on each of somites XI–XIII (thoracomeres 5–7, above second to fourth pereopods).

**Figure 1.**
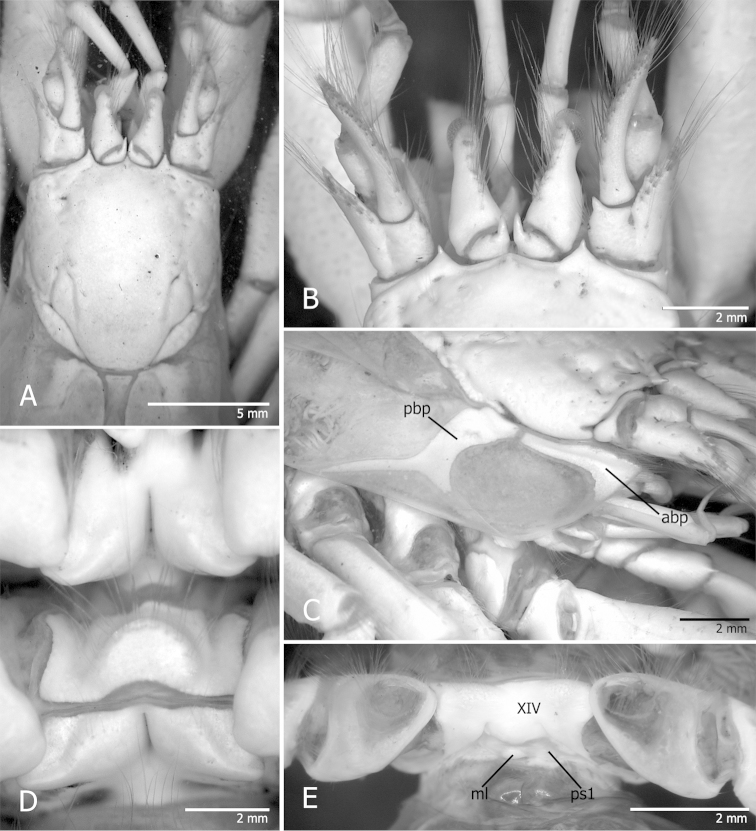
*Tomopaguropsis ahkinpechensis* sp. n.: **A** holotype male 8.9 mm (EPC201310) **B–E** paratype male 8.1 mm (USNM 12376795) **A** Shield, cephalic appendages and anterior portion of posterior carapace, dorsal **B** anterior portion of shield, and cephalic appendages, dorsal **C** right branchiostegite, and portions of shield, cephalic appendages and first to fourth pereopods **D** sternites XI and XII (second and third pereopods), ventral **E** coxae of fifth pereopods, sternite XIV (fifth pereopods), and sternite of first pleonal somite, ventral. Stippled areas in A indicate membranous condition. Abbreviations: abp, anterior branchiostegal fig; pbp, posterior branchiostegal fig; ml, calcified median lobe; ps1, first pleonal somite.

**Figure 2.**
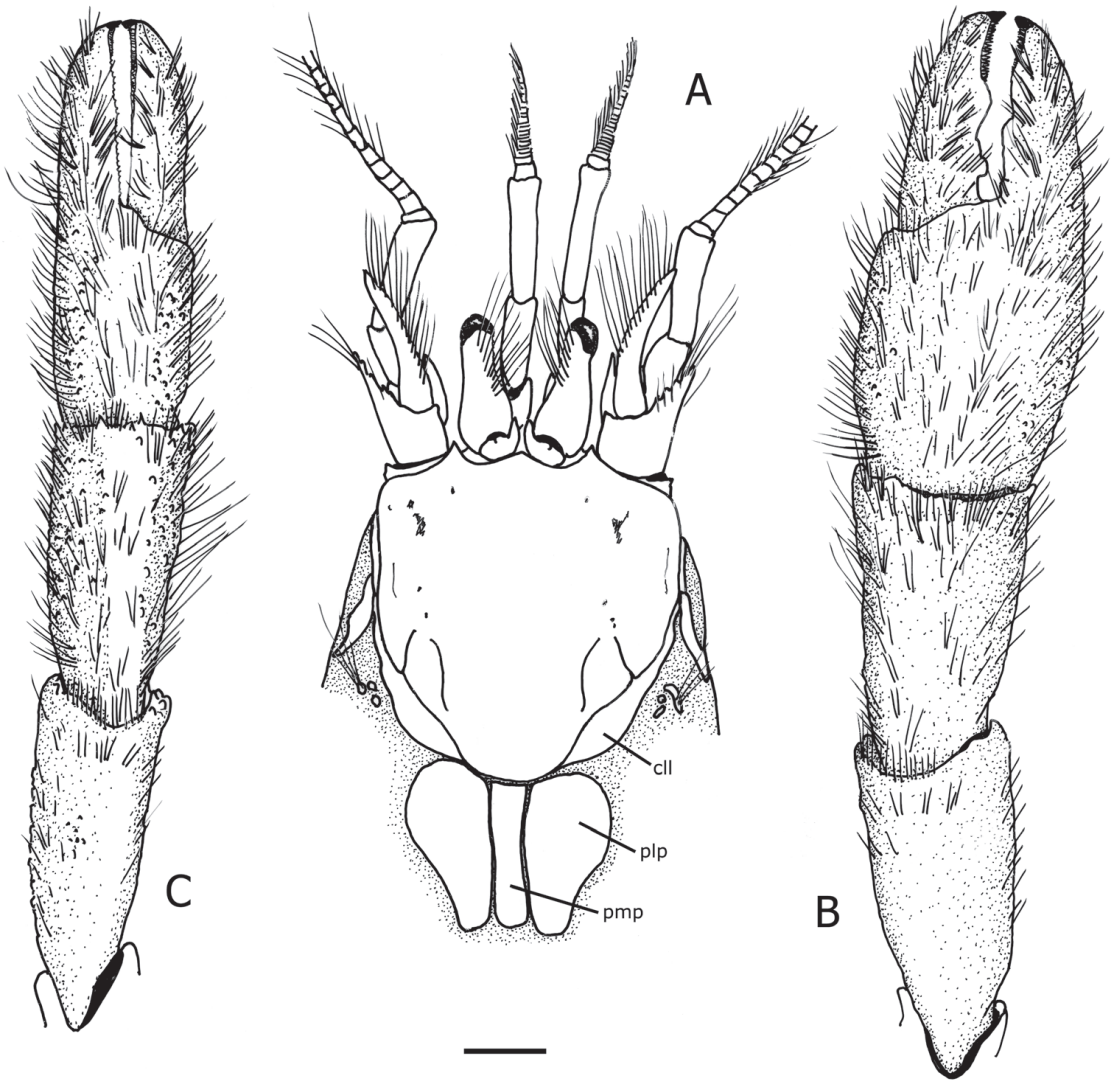
*Tomopaguropsis ahkinpechensis* sp. n. paratype male, 8.1 mm (USNM 12376795). **A** shield, cephalic appendages, and portion of posterior carapace showing dorsal figs, dorsal **B** right cheliped, dorsal **C** left cheliped, dorsal. Scale: 2 mm. Abbreviations: cll, carapace lateral lobes; pmp, posteromedian fig; plp, posterolateral figs.

Shield (Figs 1A, B, 2A) slightly longer than broad, well calcified, with short transverse rows of setae arranged longitudinally on each side from near lateral projections to near posterior margin of shield. Rostrum broadly triangular, ending roundly or in sharp spine. Anterior margins between rostrum and lateral projections concave. Lateral projections ending in sharp spine. Anterolateral margins sloping. Posterior carapace with lateral lobes (“accessory portions” of McLaughlin and Lemaitre 2001) fused to shield; with well calcified and delimited posteromedian and posterolateral figs (Figs 2A). Branchiostegite (Figs 1C, 4A) with 2 narrow calcified figs: 1 anterodorsal, and 1 posterior curving down adjacent to sulcus verticalis and bifurcated ventrally.

Ocular peduncles (Figs 1A, B, 2A) less than half length of shield, inflated basally and diminishing in width distally, with dorsomesial, longitudinal row of long setae; corneas reduced not dilated. Ocular acicles subtriangular, terminating in strong spines with mesial margins nearly contiguous.

Antennules (Fig. 2A) relatively robust, exceeding distal margin of corneas by one-fifth length of penultimate segment. Ultimate segment about one-fourth its length longer than penultimate, with scattered setae dorsally. Basal segment with strong laterodistal spine. Ventral flagellum with 8–12 articles.

Antennal peduncles (Fig. 2A) long, exceeding distal margins of corneas by full length of fifth segment. Fifth and fourth segments unarmed, sparsely setose. Third segment with strong ventromesial spine. Second segment with dorsolateral distal angle prominently produced, with long setae dorsomesially, and terminating in strong spine with 3 or 4 small spines mesially. First segment with small spine on lateral face distally. Acicle exceeding distal margin of corneas by about half length of acicle, reaching to about midpoint of fifth antennal segment; terminating in minutely bifid spine; with dorsomesial row of long bristle-like setae extending to tip of acicle. Flagellum exceeding chelae, densely setose, with setae 2–8 flagellar articles in length.

Mandible with edge of incissor process weakly sinuous, sharp, chitinous. Maxillule with external lobe slender, straight, internal lobe with 1 or 2 long bristles. Maxilla with endopodite not exceeding distal end of scaphognathite. First maxilliped with endopodite not exceeding distal margin of exopod. Second maxilliped without distinguishing characters. Third maxilliped with merus and carpus each armed with small dorsodistal spine; ischium with crista dentata consisting of about 15 small, subequal corneous teeth, with 1 accessory tooth (or as in holotype, with 2 teeth on one side); coxa with 2 sharp mesial teeth. Sternite IX (of third maxillipeds) with sharp spine on each side of midline.

Chelipeds not markedly asymmetrical, subequal in length; carpi and chela with dense, long bristle-like setae on dorsal surfaces, setae less dense and present only on distal two-thirds of ventral surfaces. Right cheliped (Fig. 2B) with fingers having numerous tufts of setae on dorsal surfaces, much less numerous on ventral surfaces, each finger terminating in inwardly curved, sharp corneous tip, armed with small blunt spines on dorsal surfaces basally and on lateral and mesial faces; cutting edges each consisting of fused corneous teeth on distal third, and 2 large, unequal rounded, calcareous teeth on proximal two-thirds, with additional smaller calcareous tooth on fixed finger. Right chela dorsal surface with scattered small spines, lateral and mesial margins rounded, with dorsomesial margin armed with irregular rows of small blunt or sharp spines; ventral face smooth. Carpus smooth except for few small blunt spines on dorsomesial margin distally, and distinct dorsodistal spine near distal margin; ventral surface smooth. Merus nearly naked, with row of setae on dorsodistal margin; smooth except for distal spine on ventromesial margin, and small, well-spaced blunt spines on ventral surface. Ischium unarmed except for ventromesial row of setae and irregular row of small blunt spines or tubercles on ventral surface.

Left cheliped (Fig. 2C) similar in setation and armature to right, except carpus with 3–5 sharp spines on dorsodistal margin.

**Figure 3.**
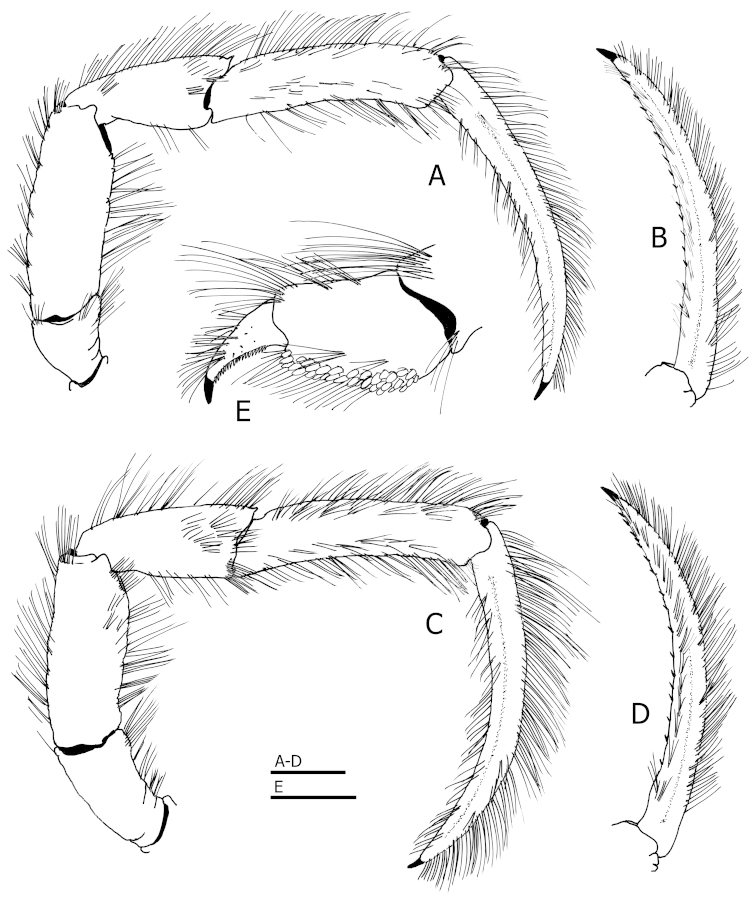
*Tomopaguropsis ahkinpechensis* sp. n. paratype male, 8.1 mm (USNM 12376795). **A** right first ambulatory leg (pereopod 2), lateral **B** dactyl of same, mesial **C** right second ambulatory leg (pereopod 3), lateral **D** dactyl of same, mesial **E** propodus and dactyl of left pereopod 4, lateral. Scales: 2 mm for **A**–**D**, 1 mm for **E**.

Ambulatory legs or pereopods 2 and 3 (Fig. 3A–D) with dense, bristle-like setae or tufts of bristle-like setae, on dorsal and ventral margins of meri, carpi, propodi and dactyls, setae arranged in short transverse rows on dorsal margins of meri, carpi and propodi, denser on propodi. Dactyls broadly curved, each about 1.8 times as long as propodi, terminating in sharp corneous claw, and armed with 12–20 small corneous spinules on ventromesial margin; with row of bristle-like setae on ventromesial margin arranged in short transverse or oblique rows. Propodi through ischium unarmed except for: bristles and small dorsodistal spine on each carpus, and also merus of first ambulatory leg (pereopod 2), irregular row of small spines on ventral margin of merus of first ambulatory leg, and row of small spines on ischium of first ambulatory leg; ischium of second ambulatory leg (pereopod 3) with ventral margin lacking spines but with row of setae.

Pereopod 4 (Fig. 3E) weakly semi-chelate, with long, bristle-like setae dorsodistally on merus, and on dorsal margins of carpus, propodus and dactyl. Dactyl subtriangular, terminating in sharp, corneous claw, lacking preungual process; with ventrolateral row of minute, fused corneous teeth. Propodal rasp consisting of 2 rows of lanceolate corneous scales distally, 3 rows proximally.

Pereopod 5 chelate. Propodal rasp well developed, occupying nearly half of lateral face of propodus.

Sternite XII (Fig. 1D) distinctly divided into anterior and anterior portions by membranous hinge. Anterior portion with semi-subcircular, setose lobe.

**Figure 4.**
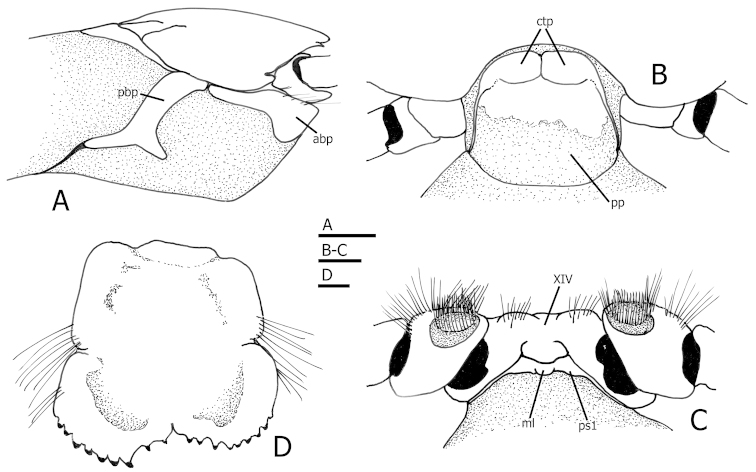
*Tomopaguropsis ahkinpechensis* sp. n. paratype male, 8.1 mm (USNM 12376795). Right side of carapace with shield, branchiostegite and anterior portion of posterior carapace, lateral **B** tergite of first pleonal somite, dorsal (up is anterior) **C** sternite XIV, coxae and gonopores of pereopods 5, and sternite of first pleonal somite (up is anterior). Abbreviations: ctp, calcareous tergal figs; pp, posterior portion; abp, pbp, ml and ps1 as in Fig. 1. Stippled areas in **A**, **B**, **C** indicate membranous condition. Scales: 2 mm for **A**, 1 mm for **B**, **C**, and 0.5 mm for **D**.

Sternite XIV (pereopod 5) weakly subdivided anteriorly into 2 setose lobes, with low posteromedian rounded ridge (Fig. 4C).

Pleon with first somite not fused to last thoracic somite. First somite with tergite consisting of pair of small calcareous figs anteriorly and partially calcified posterior portion (Fig. 4B); sternite calcified, with median lobe (Figs 1E, 4C).

Uropods strongly asymmetrical. Telson (Fig. 4D) nearly symmetrical, longer than broad; with distinct lateral indentations separating anterior and posterior portions; posterior lobes separated by small, shallow V-shaped sinus (more visible in holotype male 8.9 mm), distal margins of posterior lobes armed with small, mostly blunt spines with corneous tips.

Male with paired gonopores, each with slightly protruding vas deferens; lacking pleopods 1; pleopods 2–5 present on left side, biramous. Female unknown.

##### Etymology.

The species name is derived from the Mayan “Ah-Kin-Pech” (meaning “place of snakes and ticks”), given by that civilization to a settlement where the city of Campeche, Mexico, is now located. The Mayan name was hispanicized and used for the modern city and adjacent Campeche Bank, in the vicinity of which this new species was collected.

##### Distribution.

So far known only from the southwestern Gulf of Mexico, off the Campeche Bank; 780 to 827 m.

##### Habitat.

Gastropod shells.

##### Variations.

In the holotype male, the rostrum terminates in a sharp spine whereas in the paratype the rostrum terminates bluntly (Fig. 1A, B). The carpus of the right cheliped is more spinose than the paratype, having a dorsodistal margin armed with a distinct mesial spine and four small blunt spines laterally, the dorsomesial margin has irregular row of five distinct spines increasing in size distally and surrounded by irregularly placed small, blunt spines.

##### Affinities.

This new species is curiously more similar morphologically to a congener from Indonesian waters, *Tomopaguropsis crinita*, than to the other only known congener from the western Atlantic, *Tomopaguropsis problematica*. In the new species and *Tomopaguropsis crinita*, the ocular peduncles diminish in width distally, and corneas are reduced or weakly dilated; the chelipeds and ambulatory legs are covered with dense, long bristle-like setae most frequently arranged in tufts, but otherwise are unarmed or at most with scattered small tubercles; and males lack paired pleopods 1 (at least in the known male specimens of both species).

##### Remarks.

While this new species can be placed in *Tomopaguropsis* as currently defined (McLaughlin 1997, 2003), it is unusual and differs from other congeners in the development of calcified structures on the branchiostegite, posterior carapace, and pleon, at least based on the only two known male specimens. On the branchiostegite, all species of *Tomopaguropsis* have a narrow, calcified anterodorsal fig, but in *Tomopaguropsis ahkinpechensis* sp. n. there is also a narrow, calcified posterior fig that curves down following the sulcus verticalis and bifurcates ventrally (Figs 1C, 4A). In the new species, the posterior carapace has three calcified, well developed dorsal figs anteriorly (Fig. 2A): one dorsomedian or (“posteromedian fig”), and two dorsolateral (“posterolateral figs”). Also, in *Tomopaguropsis ahkinpechensis* sp. n., the first pleonal somite is not fused to the last thoracic somite, has a completely calcified sternite with a median lobe, and a tergite with a pair of fully calcified anterior figs and partially calcified posterior portion (Fig. 4B, C). The sternite of the first pleonal somite in *Tomopaguropsis ahkinpechensis* sp. n. is similar to that of *Tomopaguropsis crinita* in having a median lobe, but in *Tomopaguropsis crinita* the sternite is fused to the last thoracic somite and not as strongly calcified as in *Tomopaguropsis ahkinpechensis* sp. n.. The unique morphology of the branchiostegite and first pleonal somite in *Tomopaguropsis ahkinpechensis* sp. n., could well justify the placement of this new species, and possibly *Tomopaguropsis crinita* as well, in a separate new genus. Given that only two specimens of a single sex of this new species, and only four of *Tomopaguropsis crinita*, are known, it is best to wait for additional material or obtain molecular data, in order to better evaluate whether these characters reflect or not the evolution of a separate clade that could justify their placement in a new genus.

The only two known male specimens of *Tomopaguropsis ahkinpechensis* sp. n. lack pleopods 1. As noted by McLaughlin (1997), male paired pleopods 1 could possibly be a variable condition in species of *Tomopaguropsis*. McLaughlin (1997) placed her two species *Tomopaguropsis crinita* and *Tomopaguropsis miyakei* in *Tomopaguropsis*, despite the absence of paired pleopods 1 in males of the former and that males of the latter were unknown. The presence of pleopods 1 in males was one of the main characters used by Alcock (1905) when he established the genus *Tomopaguropsis* for his *Tomopaguropsis lanata* and A. Milne-Edwards and Bouvier’s (1893) Eupagurus ?problematicus (= *Tomopaguropsis problematica*, with spelling corrected). McLaughlin (1997) noted that A. Milne-Edwards and Bouvier did not mention male pleopods 1 in the description of their taxon, and presumed that Alcock’s assignment of Eupagurus ?problematicus to *Tomopaguropsis* was based on remarks by A. Milne-Edwards and Bouvier’s that implied the presence of pleopods 1 in Eupagurus ?problematicus. In order to determine whether or not males of that species had paired pleopods 1, McLaughlin (1997) examined male specimens of *Tomopaguropsis problematica* in museum collections and found them all to lack paired pleopods 1. However, we have examined one specimen (USNM 103420, 3.9 mm, from off Honduras, Caribbean Sea) which has paired pleopods 1 present and both male (paired) and female (unpaired right) gonopores, lending support to McLaughlin’s assertion that male paired pleopods 1 may be a variable condition (possibly the result of parasitic feminization) in at least two species of *Tomopaguropsis*, *Tomopaguropsis lanata*, and now *Tomopaguropsis problematica*.

There is a striking similarity in sternite XII (of third pereopods) and first pleonal sternite and tergite of *Tomopaguropsis ahkinpechensis* sp. n. with the two species of the family Pylojacquesidae (*Pylojacquesia colemani* McLaughlin & Lemaitre, 2001, and *Lemaitreopsis holmi* McLaughlin, 2007). In the new species and the two pylojacquesids, the anterior and posterior portions of sternite XII are distinctly separated by a membranous “hinge”; and the first pleonal somite has a pair of calcified figs anteriorly on the tergite, and a median lobe (in *Tomopaguropsis ahkinpechensis* sp. n.) or posteriorly directed projection (in Pylojacquesidae). The similarity of these unusual characters conceivably can be interpreted as evidence of a close shared ancestry between *Tomopaguropsis*, as a member of the Paguridae, and the Pylojacquesidae, a phylogenetic relationship previously suggested by McLaughlin et al. (2007) and McLaughlin (2007). A full phylogenetic evaluation of *Tomopaguropsis* and its five species, however, must await the study and discovery of additional specimens, ideally combined with genetic analyses.

When McLaughlin (2003) updated the generic diagnosis of *Tomopaguropsis* she stated that a preungual process was absent in species of this genus, even though she (McLaughlin 1997) had previously reported the presence of a prominent preungual process in *Tomopaguropsis miyakei*. Furthermore, examination of USNM specimens has shown that a preungual process is also present in *Tomopaguropsis problematica*.

### Key to species of *Tomopaguropsis*

**Table:** 

1	Ocular peduncles decreasing in width distally, corneas not dilated; antennal peduncles distinctly exceeding distal margins of corneas by one-third or more length of fifth antennal segment	**3**
–	Ocular peduncles not decreasing in width distally, subcylindrical, corneas moderately dilated; antennal peduncles not exceeding or at most slightly exceeding distal margins of corneas	**2**
2	Ventral margins of dactyls of ambulatory legs (pereopods 2, 3) armed with corneous spines; posterior lobes of telson nearly symmetrical, separated by V-shaped median cleft	***Tomopaguropsis miyakei* McLaughlin, 1997** (Indonesia)
–	Ventral margins of dactyls of ambulatory legs (pereopods 2, 3) unarmed, lacking corneous spines; posterior lobes of telson asymmetrical, separated by narrow slit	***Tomopaguropsis problematica* (A. Milne-Edwards & Bouvier, 1893)** (Western Atlantic)
3	Dorsal surfaces of chelae distinctly spinulose	***Tomopaguropsis lanata* Alcock, 1905** (Indian Ocean)
–	Dorsal surfaces of chelae not spinulose or at most with scattered small tubercles	**4**
4	Branchiostegite with narrow, calcified posterior fig curving down, following sulcus verticalis, and bifurcated ventrally (Figs 1C, 4A); propodus of pereopod 4 longer than high (Fig. 3E)	***Tomopaguropsis ahkinpechensis* sp. n.** (Gulf of Mexico)
–	Branchiostegite lacking narrow, calcified posterior fig; propodus of pereopod 4 about as long as high	***Tomopaguropsis crinita* McLaughlin, 1997** (Indonesia)

## Supplementary Material

XML Treatment for Tomopaguropsis ahkinpechensis
